# Time-Lag of Seasonal Effects of Extreme Climate Events on Grassland Productivity Across an Altitudinal Gradient in Tajikistan

**DOI:** 10.3390/plants14081266

**Published:** 2025-04-21

**Authors:** Yixin Geng, Hikmat Hisoriev, Guangyu Wang, Xuexi Ma, Lianlian Fan, Okhonniyozov Mekhrovar, Madaminov Abdullo, Jiangyue Li, Yaoming Li

**Affiliations:** 1 Key Laboratory of Ecological Safety and Sustainable Development in Arid Lands, Xinjiang Institute of Ecology and Geography, Chinese Academy of Sciences, Urumqi 830011, China; gengyixin23@mails.ucas.ac.cn (Y.G.); wangguangyu19@mails.ucas.ac.cn (G.W.); maxx@ms.xjb.ac.cn (X.M.); flianlian@ms.xjb.ac.cn (L.F.); okhonniyozov.mekhrovar@gmail.com (O.M.); 2 Research Center for Ecology and Environment of Central Asia, Chinese Academy of Sciences, Urumqi 830011, China; 3 University of Chinese Academy of Sciences, Beijing 100049, China; 4 Institute of Botany, Plant Physiology and Genetics of Tajikistan Academy of Sciences, Dushanbe 734002, Tajikistan; hhikmat@mail.ru (H.H.); asrorijon@mail.ru (M.A.)

**Keywords:** seasonal extreme climate, grasslands, net primary productivity, lag effects, Tajikistan

## Abstract

Mountain grassland ecosystems around the globe are highly sensitive to seasonal extreme climate events, which thus highlights the critical importance of understanding how such events have affected vegetation dynamics over recent decades. However, research on the time-lag of the effects of seasonal extreme climate events on vegetation has been sparse. This study focuses on Tajikistan, which is characterized by a typical alpine meadow–steppe ecosystem, as the research area. The net primary productivity (NPP) values of Tajikistan’s grasslands from 2001 to 2022 were estimated using the Carnegie–Ames–Stanford Approach (CASA) model. In addition, 20 extreme climate indices (including 11 extreme temperature indices and 9 extreme precipitation indices) were calculated. The spatiotemporal distribution characteristics of the grassland NPP and these extreme climate indices were further analyzed. Using geographic detector methods, the impact factors of extreme climate indices on grassland NPP were identified along a gradient of different altitudinal bands in Tajikistan. Additionally, a time-lag analysis was conducted to reveal the lag time of the effects of extreme climate indices on grassland NPP across different elevation levels. The results revealed that grassland NPP in Tajikistan exhibited a slight upward trend of 0.01 gC/(m^2^·a) from 2001 to 2022. During this period, extreme temperature indices generally showed an increasing trend, while extreme precipitation indices displayed a declining trend. Notably, extreme precipitation indices had a significant impact on grassland NPP, with the interaction between Precipitation anomaly (PA) and Max Tmax (TXx) exerting the most pronounced influence on the spatial variation of grassland NPP (q = 0.53). Additionally, it was found that the effect of extreme climate events on grassland NPP had no time-lag at altitudes below 500 m. In contrast, in mid-altitude regions (1000–3000 m), the effect of PA on grassland NPP had a significant time-lag of two months (*p* < 0.05). Knowing the lag times until the effects of seasonal extreme climate events on grassland NPP will appear in Tajikistan provides valuable insight for those developing adaptive management and restoration strategies under current seasonal extreme climate conditions.

## 1. Introduction

With the intensification of global climate change, the intensity, frequency, and persistence of seasonal extreme climate events have all increased, thereby profoundly impacting vegetation dynamics in terrestrial ecosystems [1,2,3]. For example, Li et al. [4] observed that vegetation in desert steppe and steppe desert exhibited relatively low sensitivity to extreme temperature indices during summer, whereas forests and sand deserts generally showed minimal responsiveness to extreme precipitation indices in the same season. Similarly, an analysis of the Tarim Basin (2000–2022) revealed that the synergistic effects of extreme climate indices exert a more pronounced influence on ecosystem quality (EQI) than individual climatic extremes, with particularly strong impacts observed in forest and shrubland ecosystems [5]. The effects of seasonal extreme climate events on vegetation growth are often complex and are typically asymmetrical, only appearing after a certain time-lag [6,7]. These lags in effects arise when extreme climatic conditions exceed the tolerance thresholds of vegetation, thereby delaying the growth response to such variables [8]. Recent studies demonstrate that in Central Asia’s land surface phenology, the frequency of warm extremes (TX90p) serves as the primary determinant of both the end of the season (EOS) and the length of the growing season (LOS), with cold extremes (TX10p) and mean temperature (TXmean) exhibiting secondary and tertiary influences, respectively [9]. For instance, Ma et al. [10] revealed that extreme climate indices have an immediate (0-month, no lag) effect on vegetation coverage in northern China. Similarly, in their study of the ecologically sensitive regions of Inner Mongolia, Yuan et al. [11] found that vegetative responses to seasonal extreme climate events can also exhibit temporal delays. Specifically, the extreme precipitation index RX5day was significantly associated with an effect on vegetation growth that only appeared two months later. Understanding the lag time of the effects of seasonal extreme climate events on vegetation growth is essential for gaining insight into the vulnerabilities of terrestrial ecosystems to climate change. This knowledge can inform strategies to enhance ecosystem resilience in the face of increasingly common climatic extremes.

Alpine grasslands are a vital component of global terrestrial ecosystems, constituting the dominant vegetation type and covering approximately two-thirds of high plateaus. These ecosystems are characterized by unique climatic features, including intense solar radiation, extended sunlight duration, lower temperatures and air pressure, and distinct seasonal and spatial precipitation variability [12,13]. Extensive research has demonstrated that alpine grasslands are highly sensitive to climate change. For instance, drought has been shown to have a significant temporal impact on the growing season vegetation of the Tibetan Plateau, with its effects peaking in July and August [14]. The Kobresia meadow area of the Qinghai-Tibetan Plateau exhibits a general trend toward increasing warm extremes, with more pronounced warming occurring during nighttime. However, the absolute magnitude of increase in maximum daytime temperatures exceeds that of nighttime temperatures [15]. Yan et al. [16] revealed that climate warming can stabilize alpine ecosystems facing extreme precipitation events by altering vegetation species composition. Similarly, An et al. [17] highlighted that the gradual increase in extremely high temperatures and precipitation in Qinghai Province has led to grassland degradation. In the Qilian Mountain region, the fastest warming and the mildest wetting trends, which have been observed at both the lowest elevations (<3200 m) and the highest elevations (>4200 m), are likely to heighten the vulnerability of vegetation to drought [18]. While existing studies indicate that alpine grasslands respond significantly to seasonal extreme climate events, the time-lag of the effects remains poorly understood.

Tajikistan, located in the heart of the Eurasian continent, has a high-altitude grassland ecosystem that accounts for approximately 30% of its total area. Globally, alpine grasslands constitute only about 10–15% of the total grassland area, according to the IPCC [19]. This suggests that Tajikistan has a disproportionately high share of alpine grasslands, making it an outlier in terms of high-altitude pasture coverage. This habitat type is dominant in the country, providing grazing grounds for local pastoralism. However, in recent years, the region has experienced significant degradation in its *Artemisia* steppe due to rising temperatures and increasingly frequent seasonal extreme climate events, which have severely affected pasture yields and economic development. For instance, Chen et al. [20] analyzed the relative impacts of climate change and human activities on the Central Asian arid and semiarid steppes between 1982 and 2015. They found that precipitation is the primary climatic factor influencing grassland changes in much of Central Asia. A decrease in precipitation leads to reduced photosynthetic efficiency, inhibited vegetation activity, and a decline in organic matter, thereby suppressing grassland growth. Luo et al. [21] explored the impact of extreme climate indices on vegetation dynamics in Central Asia and revealed that the region, which is composed of mainly mountainous areas, is experiencing an increasing frequency of seasonal extreme climate events. Gao et al. [22] studied the effects of seasonal extreme climate on the autumn phenology of Central Asian grasslands, finding that extreme precipitation and heat waves have led to a general advancement of autumn phenology in the region’s grassland vegetation. Hu et al. [23] analyzed the alpine grassland regions of Central Asia and observed an increasing trend in warm nights and warm days that was accompanied by a decline in cold nights and cool days. These changes have directly led to an extended growing season and have thus exerted a positive influence on grassland vegetation productivity. However, research on the time-lag of seasonal extreme climate events on grassland vegetation growth at different altitudes in Tajikistan remains scarce, thereby posing a significant obstacle to developing effective grassland management strategies and enhancing resilience to seasonal extreme climate risks.

In this study, we utilized meteorological data with grassland vegetation net primary productivity (NPP) to investigate the impacts of seasonal extreme climate events on grassland vegetation NPP across various altitudinal bands in Tajikistan, focusing on lagging responses. Our specific objectives were as follows: (1) to evaluate the spatiotemporal distribution patterns of alpine grassland vegetation NPP and extreme climate indices in Tajikistan; (2) to identify the single-factor and dual-factor interactions of extreme climate indices influencing grassland vegetation NPP dynamics; and (3) to analyze the lag time of the effects of extreme climate indices on grassland NPP in Tajikistan. Therefore, this study is of great significance for understanding the mechanisms of vegetation response to seasonal extreme climate events and for providing scientific guidance in ecosystem management and sustainable development in the region.

## 2. Materials and Methods

### 2.1. Study Area

**Figure 1:**
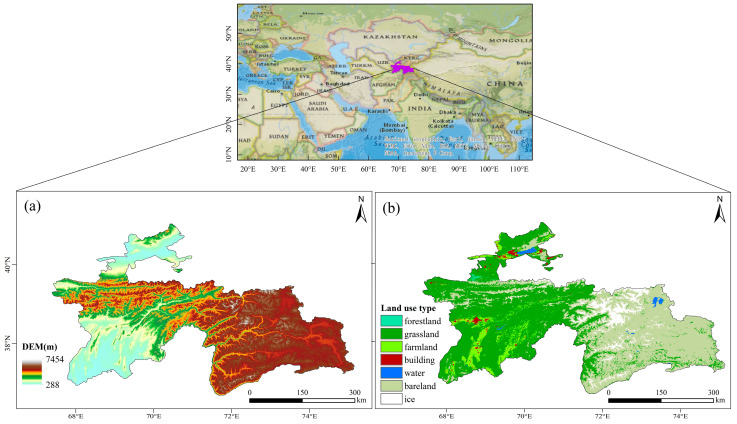
Location of Tajikistan. (**a**) Topography of Tajikistan using the digital elevation model; (**b**) land cover map of Tajikistan. Drawing review No.: GS(2020)4399.

Tajikistan (67°31′–75°14′ E, 36°40′–41°05′ N) is a mountainous country located in the heart of the Eurasian continent, with more than 50% of its territory at an elevation exceeding 3000 m. The country’s mountainous and plateau areas account for approximately 93% of its total land area, about 143,000 km^2^. The region experiences a continental climate with scarce precipitation, hot summers, and cold winters. The annual average temperature ranges from 0.1 °C to 3.5 °C, and the annual precipitation varies from 102.7 mm to 748.8 mm, with the maximum-to-minimum precipitation ratio being approximately 7:1, which makes the area prone to frequent seasonal extreme climate events. Tajikistan’s climate is strongly influenced by its topography, which has resulted in subtropical, temperate, and subarctic climatic conditions. The elevation ranges from 288 to 7495 m, thus creating significant topographical variation. The primary land use type is grassland, which supports a diverse range of herbaceous vegetation, including grasses, ryegrass (*Lolium perenne* L.), and sedges (*Carex* L.). These grasslands are of significant value to local livestock farming and play a crucial role in the country’s agriculture and economy (Figure 1).

### 2.2. Data Sources

The monthly climate data utilized in this research, including measures of precipitation, temperature, and radiation, were obtained from the TerraClimate dataset. This dataset features a spatial resolution of 4 km and spans the years 2001 to 2022. The extreme climate indices, encompassing daily maximum and minimum temperatures as well as daily precipitation, were obtained from the ERA5 dataset. This dataset has a spatial resolution of 10 km and a temporal scale of daily data, covering the period from 2001 to 2022. We accessed the “ECMWF/ERA5_LAND/DAILY_AGGR” dataset using the GEE platform for maximum and minimum temperature data. The temperature values in Kelvin (K) were converted to Celsius (°C), and the precipitation values, originally in meters (m), were converted to millimeters (mm).

The NDVI data utilized in this study were obtained from the MOD13Q1 dataset, which features a spatial resolution of 250 m and a temporal scale of 16 days. All NDVI datasets spanning the period from 2001 to 2022 were pre-processed on the GEE platform. Monthly NDVI values were generated using the maximum value composite method, and the spatial resolution was standardized to 500 m through resampling to facilitate overlay analyses.

Land cover information was obtained from the MCD12Q1 dataset, which provides annual data at a spatial resolution of 500 m for the period from 2001 to 2022.

Elevation data were obtained from the GDEMV2 dataset, which has a spatial resolution of 30 m and spans from 2001 to 2022.

### 2.3. Research Methods

**Figure 2:**
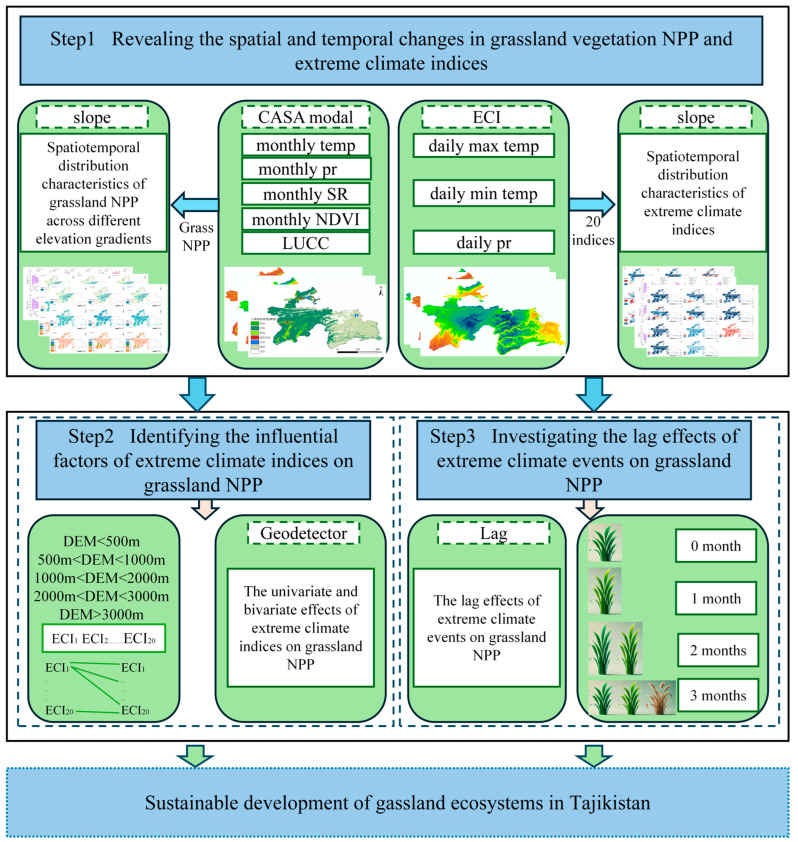
Overview of methodology.

The research flowchart is shown in Figure 2. This study aimed to evaluate the response of grassland NPP in Tajikistan to seasonal extreme climate events, and the research process involved several key steps. First, the grassland vegetation NPP was estimated using the CASA model and stochastic regression models, which were followed by a spatiotemporal analysis to reveal the spatial and temporal distribution patterns of grassland NPP. Next, 20 extreme climate indices were calculated and analyzed to explore their relationship with grassland NPP, with a particular focus on the impact of individual climate factors and the interactions of multiple climate factors on the dynamics of grassland NPP. Finally, the delayed effects of seasonal extreme climate events were assessed to analyze the impacts of climate change on grassland NPP.

#### 2.3.1. Simulation of NPP Based on CASA Model

The Carnegie–Ames–Stanford Approach (CASA) model estimates the net primary productivity (NPP) of terrestrial ecosystems by leveraging NDVI data and the principles of light energy utilization. In this study, precipitation, temperature, radiation, NDVI, and land use data from 2001 to 2022 were integrated to model the NPP dynamics of the study region using the CASA model. The specific calculation steps are as follows [24,25]:

(1) NPP(x,t)=APAR(x,t)×ε(x,t)

APAR represents the absorbed photosynthetically active radiation by vegetation; x refers to the corresponding grid cell, and t denotes the corresponding month. The calculation methods for the relevant parameters are as follows:

(2) APAR(x,t)=PAR(x,t)×FPAR(x,t)

(3) $$FPAR(x,t)={\left(FPAR(x,t\right)}_{NDVI}+{FPAR(x,t)}_{SN})/2$$

(4) $${FPAR(x,t)}_{NDVI}=\frac{\left(NDVI(x,t)-NDVI_{i,\min})\times (FPAR_{\max}-FPAR_{\min}\right)}{NDVI_{i,\max}-NDVI_{i,\min}}+FPAR_{\min}$$

(5) $${FPAR(x,t)}_{RVI}=\frac{\left(RVI(x,t)-RVI_{i,\min})\times (FPAR_{\max}-FPAR_{\min}\right)}{RVI_{i,\max}-RVI_{i,\min}}+FPAR_{\min}$$

(6) $$RVI(x,t)=\frac{1+NDVI(x,t)}{1-NDVI(x,t)}$$

*PAR* constitutes 50% of the total downward shortwave radiation. For a given grid cell x and month t, *NDVI(x,t)* and *RVI(x,t)* denote the normalized difference vegetation index and the ratio vegetation index, respectively. The fractions of absorbed *PAR (FPAR)* by the vegetation layer, expressed as *FPAR(x,t)_NDVI_* and *FPAR(x,t)_RVI_*, are quantified using *NDVI* and *RVI* for the corresponding spatial and temporal scales. The upper and lower bounds of *FPAR* are parameterized as 0.95 and 0.001, respectively. For the i-th vegetation type, *NDVI_i,max_* and *NDVI_i,min_* represent its maximum and minimum values, while *RVI_i,max_* and *RVI_i,min_* correspond to the 95th and 5th percentiles of all the observed *RVI* data.

#### 2.3.2. Description of Extreme Climate Indices

This study uses Python package “pyclimdex” (Version: 0.0.1) to calculate extreme climate indices based on daily meteorological data from Tajikistan. These indices describe seasonal extreme climate conditions that can occur multiple times within a year, rather than once every few years. They are widely used and have been easy to interpret in previous studies [26,27]. The 20 extreme climate indices recommended by the Expert Team on Climate Change Detection and Indices (ETCCDMI) were calculated here. There were 9 precipitation-based indices and 11 temperature-based indices. Their specific definitions are presented in Table 1.

#### 2.3.3. Slope Analysis

To examine the linear trends of annual mean grassland NPP and extreme climate indices in Tajikistan, a univariate linear regression approach was employed. The formula is as follows [28]:

(7) $$S=\frac{n\sum_{i=1}^{n}i\times x_{i}-\sum_{i=1}^{n}i\sum_{i=1}^{n}x_{i}}{n\sum_{i=1}^{n}i^{2}-(\sum_{i=1}^{n}i{)}^{2}}$$

*S* denotes the slope of the linear trend, with n representing the length of the time series (n = 22). *S* > 0 indicates an increasing trend in *x*, while *S* < 0 signifies a declining trend.

#### 2.3.4. Coefficient of Variation

The coefficient of variation (CV) reflects the degree of variation in a variable over time and is used to assess the stability of the extreme climate indices in Tajikistan from 2001 to 2022. The formula is as follows [29]:

(8) $$CV=\frac{\sigma }{\bar{N}}$$

where *σ* represents the standard deviation of the variable, and *μ* is the mean value of the 20 extreme climate indices from 2001 to 2022. A larger CV value indicates greater instability, while a smaller CV value suggests greater stability.

#### 2.3.5. Geographical Detector

The geographical detector is a novel statistical method for detecting spatial differentiation and revealing its driving factors [30,31]. The geographical detector includes four detectors. This study used the differentiation, factor, and interaction detection detectors.

Differentiation and factor detection: This detects the spatial differentiation of y (grassland variable NPP) and the degree to which factor x explains the spatial differentiation of attribute y. The q value is used to measure this, and the formula is as follows:

(9) $$q=1-\frac{\sum_{h=1}^{L}N_{h}{\delta_{h}}^{2}}{N\delta^{2}}=1-\frac{SSW}{SST}$$

(10) $$SSW=\sum_{h=1}^{L}N_{h}{\delta_{h}}^{2},SST=N\delta^{2}$$

*h* = 1, 2, …, *L* denote the number of layers for factor x. *N* represents the total number of layers across the entire region, and *N_h_* corresponds to the number of layers in the h-th category. The terms *δ_h_^2^* and *δ^2^* refer to the variance of the seasonal extreme climate factors and the associated dependent variable NPP in grasslands, respectively. *SSW* and *SST* represent the sum of squares within groups and the total sum of squares, respectively. The value of q lies in the range [0, 1], where a higher value of q indicates a stronger explanatory power of the independent variable x for NPP.

Interaction detection: This process examines the interplay between factors, assessing whether the combined influence of x_1_ and x_2_ enhances or diminishes their capacity to explain variation in the dependent variable y. The procedure begins by independently calculating the q values for each factor, q(x_1_) and q(x_2_). Subsequently, the interaction q value, q(x_1_ ∩ x_2_), is determined. A comparative analysis is then performed to evaluate the relative contributions of q(x_1_ ∩ x_2_) against q(x_1_) and q(x_2_). The relationship between these factors is clearly visualized in Table 2.

#### 2.3.6. Lag Effect Analysis

A time-lag indicates an inability of vegetation to respond promptly to dynamic changes in the factors influencing its growth. This study employed the Pearson correlation coefficient to quantify the lag in the effects of various extreme climate indices on grassland NPP across different elevation bands. When the correlation coefficient r_i_ reaches its maximum value, this indicates that the optimal correlation has been achieved. The corresponding month i represents the optimal time-lag [32,33].

(11) $$\begin{array}{l} r_{i}=corr(x,Y_{i})\\ R_{\max\_\log}=\max(r_{i}) \end{array}\;0\leq i\leq 3$$

*i* represents a lag of i months, where *x* is the monthly time series of grassland NPP at different elevation bands from 2001 to 2022, and *Y* refers to the extreme climate indices. *Y_i_* is the monthly time series of extreme climate indices with a lag of *i* months, and *r_i_* is the Pearson correlation coefficient for the lag of *i* months.

## 3. Results

### 3.1. Spatiotemporal Variations in Grassland NPP

**Figure 3:**
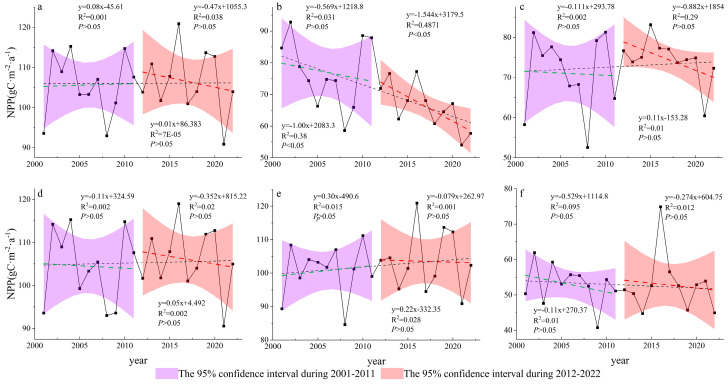
Changes in annual grassland NPP across different elevation gradients in Tajikistan during 2001–2022. (**a**) Entire Tajikistan; (**b**) elevation <500 m; (**c**) elevation 500–1000 m; (**d**) elevation 1000–2000 m; (**e**) elevation 2000–3000 m; and (**f**) elevation >3000 m. Green line, the grassland NPP change trend from 2001 to 2011; red line, the grassland NPP change trend from 2012 to 2022; black line, the grassland NPP change trend from 2001 to 2022.

The changes in annual average grassland NPP across different elevation bands are illustrated in Figure 3b–f. Regions with elevations below 500 m and above 3000 m showed a declining trend, with the <500 m elevation region experiencing a significant decrease. In contrast, areas at elevations between 500 m and 3000 m displayed an increasing trend. Among these bands, the 1000–2000 m elevation range had the highest average annual grassland NPP at 105.26 gC/(m^2^·a), followed by the 2000–3000 m and 500–1000 m ranges, with values of 102.14 gC/(m^2^·a) and 72.58 gC/(m^2^·a), respectively. Regions below 500 m and above 3000 m had relatively low average annual grassland NPP values, with 71.58 gC/(m^2^·a) and 52.85 gC/(m^2^·a), respectively. Ranked from highest to lowest, the rates of change in annual average grassland NPP across elevation bands were as follows: 2000–3000 m [0.22 gC/(m^2^·a)] > 500–1000 m [0.11 gC/(m^2^·a)] > 1000–2000 m [0.05 gC/(m^2^·a)] > 293–500 m [−1.00 gC/(m^2^·a)] > 3000–7454 m [−0.11 gC/(m^2^·a)]. Notably, the <500 m region experienced a significant decline in grassland NPP between 2012 and 2022.

It is crucial to recognize that the estimation of grassland net primary productivity (NPP) in this study is inherently affected by uncertainties associated with the maximum light energy utilization (ε) parameter in the model. Through a comprehensive sensitivity analysis, we varied the value of ε within the range of 0.8 to 1.1. The results revealed that the minimum and maximum error percentages of the NPP estimates were 0% and 10%, respectively. This pronounced variation indicates that the model’s output is highly sensitive to fluctuations in the ε parameter, underscoring the need for accurate parameter calibration in future research.

**Figure 4:**
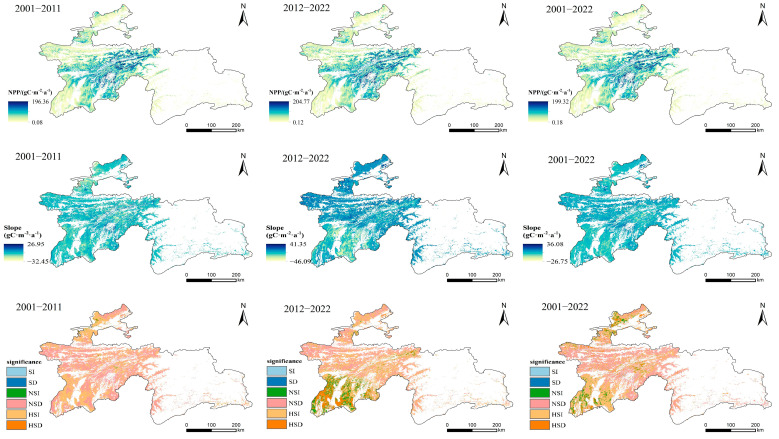
Spatial distribution of grassland NPP in Tajikistan across different altitude gradients from 2001 to 2022. SI, significantly increased; SD, significantly decreased; NSI, non-significantly increased; NSD, non-significantly decreased; HSI, highly significantly increased; and HSD, highly significantly decreased.

The annual average grassland NPP in Tajikistan over the past 22 years followed an uneven distribution. High-value areas were primarily located in the central and the northern parts of the Khatlon region, with most grassland NPP exceeding 70 gC/(m^2^·a) and with a few areas exceeding 150 gC/(m^2^·a). Low-value areas were mainly found in the southern parts of Khatlon and the plateau and gully regions of the Gorno-Badakhshan Autonomous Region. The regions that experienced a significant increase in grassland NPP from 2001 to 2022 were primarily concentrated in the southwestern and northern plain areas, with sporadic zones showing non-significant increases. The most significant increases were found in the high-altitude central regions. Specifically, between 2001 and 2011, the grassland NPP in Tajikistan generally showed either a very significant increase or a non-significant decrease, with a few non-significant increase trends scattered in the central mountainous regions. In contrast, the spatial pattern of NPP change became more complex from 2012 to 2022, with the southwestern low-altitude areas showing very significant decreases and non-significant increases, while the northern low-altitude areas predominantly showed very significant increases (Figure 4).

### 3.2. Spatiotemporal Variations in Extreme Climate Indices

**Figure 5:**
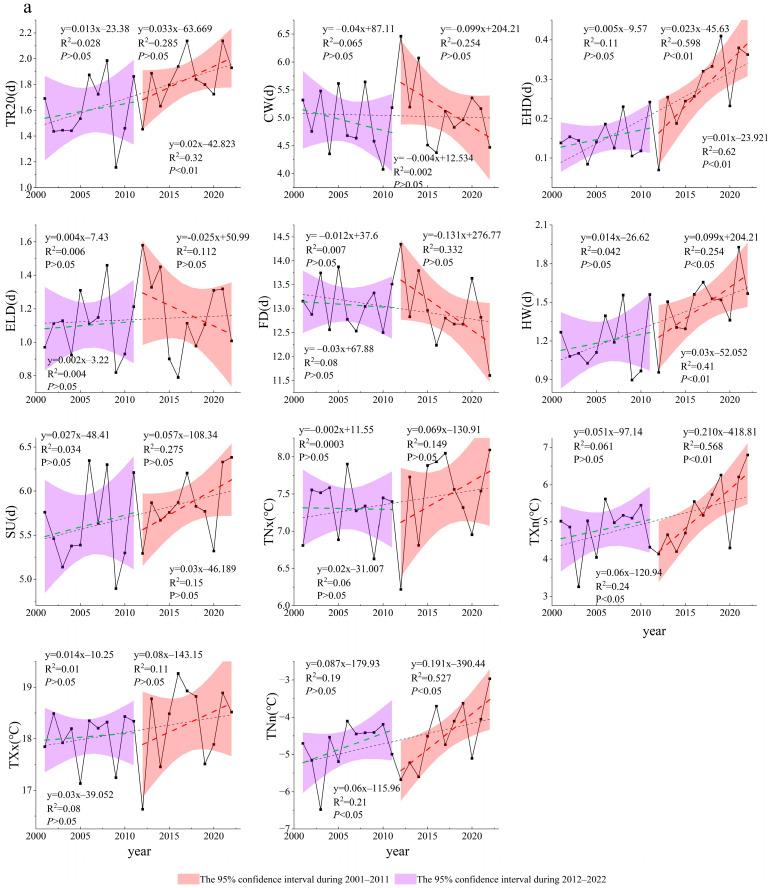

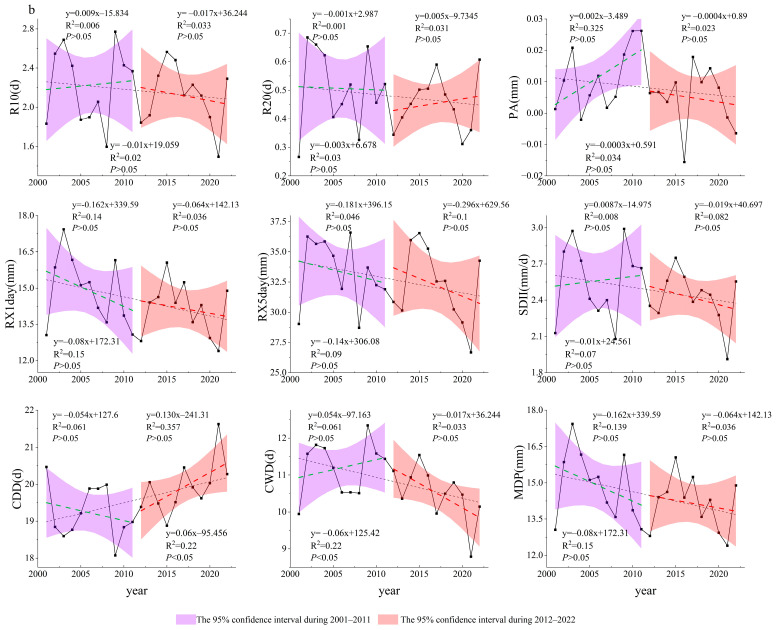
Changes in annual extreme temperature indices (**a**) and extreme precipitation indices (**b**) during 2001 to 2022. Green line, the grassland NPP change trend from 2001 to 2011; red line, the grassland NPP change trend from 2012 to 2022; black line, the grassland NPP change trend from 2001 to 2022.

Most extreme temperature indices (Figure 5a) showed an upward trend, with only FD exhibiting a downward trend. Of the indices, TXn showed the fastest increase at a rate of 0.06 °C per year (*p* < 0.05), followed by TNn, which also increased at a rate of 0.06 °C per year (*p* < 0.05). TR20 and HW both showed highly significant upward trends (*p* < 0.01), with rates of increase at 0.02 days per year and 0.03 days per year, respectively. In contrast, FD decreased at a rate of −0.03 days per year. This indicates a rise in extreme temperatures in Tajikistan as well as an increase in the frequency of extreme heat events. Of the extreme precipitation indices (Figure 5b), only CDD showed an upward trend, with a significant increase at a rate of 0.06 days per year (*p* < 0.05). Additionally, RX1day showed the fastest decrease, at a rate of −0.08 mm per year. Both CWD indices exhibited significant downward trends (*p* < 0.05), with a rate of decline of −0.06 days per year. This suggests that the number of precipitation days per year in Tajikistan is on the decline, which ultimately indicates a shift towards a hotter and drier climate.

From 2001 to 2011, the extreme climate indices were not statistically significant. Only the extreme temperature indices TNx, CW, and FD showed a downward trend with CW decreasing most significantly at a rate of −0.04 d/year. Most extreme precipitation indices showed a downward trend, with only R10, PA, CWD, and SDII increasing at rates of 0.009 d/year, 0.001 mm/year, 0.001 mm/d/year, and 0.008 d/year, respectively. From 2012 to 2022, the extreme temperature indices EHD and TXn showed a highly significant upward trend, with rates of increase of 0.02 d/year and 0.21 °C/year (*p* < 0.01), respectively. HW and TNn also showed significant upward trends, with rates of increase of 0.1 d/year and 0.19 °C/year (*p* < 0.05). Most extreme precipitation indices showed a downward trend, with only R20 and CDD increasing.

**Figure 6:**
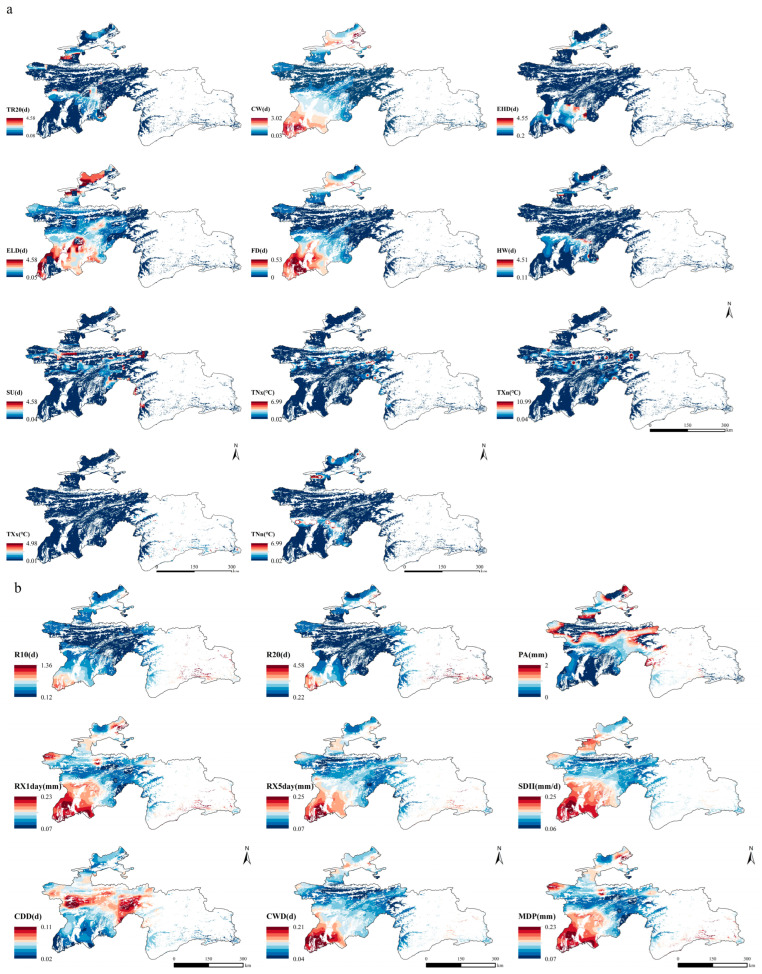
Spatial distribution of coefficients of variation of extreme temperature (**a**) and extreme precipitation (**b**) in Tajikistan during 2001–2022.

Using the coefficient of variation (CV) to analyze the spatial variability of extreme climate indices in Tajikistan (Figure 6), we found that extreme temperature indices (CW, FD, and ELD) exhibited significant fluctuation in areas below 1000 m in elevation, with CV values exceeding 0.2. This indicates that extreme temperature variations are more pronounced in low-altitude regions. In contrast, these indices showed much smaller variability in mid- to high-altitude areas, with CV values typically below 0.1, which suggests a more stable climate in these regions.

Additionally, extreme precipitation indices (RX1day, RX5day, SDII, CWD, and MDP) also demonstrated considerable spatial variability in areas below 1000 m, with CV values exceeding 0.2. This highlights the substantial spatial variation in extreme precipitation events in low-altitude regions that is likely influenced by local climate and topographic factors. Interestingly, the CDD index showed the most significant variability in the 2000–3000 m altitude range, where CV values also exceeded 0.2, indicating pronounced changes in dry conditions in this region. Furthermore, the PA index exhibited a high degree of variability in areas above 3000 m, with CV values greater than 0.2.

### 3.3. Detection of Factor Influence

**Figure 7:**
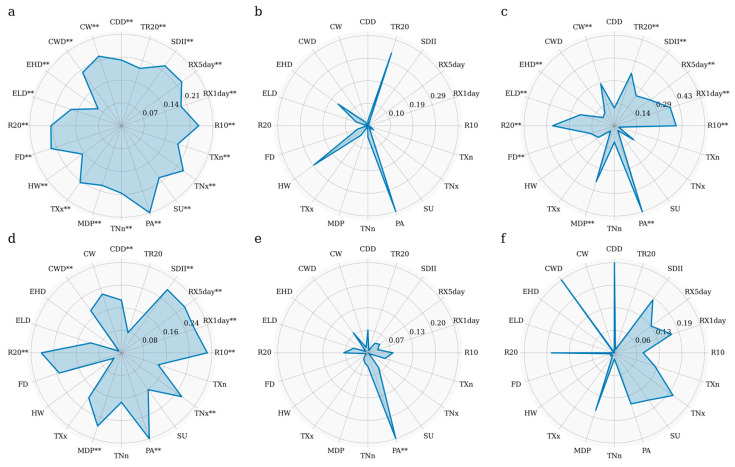
The contribution rate (q value) of extreme climate indices to NPP in different altitude gradients. (**a**) Entire Tajikistan; (**b**) elevation <500 m; (**c**) elevation 500–1000 m; (**d**) elevation 1000–2000 m; (**e**) elevation 2000–3000 m; and (**f**) elevation >3000 m; ** indicates that the *p*-value satisfies the significance threshold of *p*  <  0.01.

Factor detection (q values) results reveal the explanatory power of vegetation NPP of extreme climate indices (Figure 7a). The factors influencing the spatial variation in grassland NPP in Tajikistan were ranked by their q values as follows: PA (0.28) > R10 (0.24) > TNx (0.24) > RX5day (0.23) > SDII (0.23) > FD (0.23) > CW (0.22) > R20 (0.22) > TXx (0.22) > TNn (0.21) > CWD (0.20) > CDD (0.20) > SU (0.20) > MDP (0.19) > RX1day (0.19) > TR20 (0.19) > TXn (0.18) > ELD (0.16) > HW (0.15) > EHD (0.09). PA is the primary factor influencing the variation in grassland NPP in Tajikistan, with an explanatory power of 0.28. However, EHD has a low explanatory power of less than 0.1. Furthermore, the results for all influencing factors are significant (*p* < 0.01).

As shown in Figure 7b–f, the dominant factors influencing the spatial differentiation of grassland NPP differed across various altitude bands. In areas below 3000 m, PA was the primary influencing factor, with its explanatory power being most significant at 500–1000 m (0.58) and at 293–500 m (0.39). In regions above 3000 m, CWD was the most dominant factor with an explanatory power of 0.26. CW had the least explanatory power for grassland NPP changes in areas below 500 m and above 3000 m, with values under 0.002.

**Figure 8:**
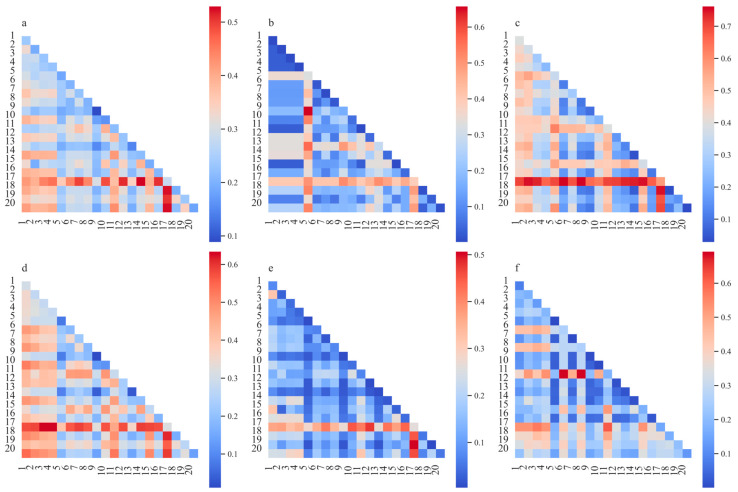
Analysis of interaction between factors. 1: R10, 2: RX1day, 3: RX5day, 4: SDII, 5: TR20, 6: CDD, 7: CW, 8: CWD, 9: EHD, 10: ELD, 11: R20, 12: FD, 13: HW, 14: TXx, 15: MDP, 16: TNn, 17: PA, 18: SU, 19: TNx, and 20: TXn. (**a**) Entire Tajikistan; (**b**) elevation <500 m; (**c**) elevation 500–1000 m; (**d**) elevation 1000–2000 m; (**e**) elevation 2000–3000 m; and (**f**) elevation >3000 m.

The interaction test was conducted to evaluate how the interplay between seasonal extreme climate factors affected NPP. The combined effects of two seasonal extreme climate factors on NPP are highlighted in Figure 8. The interaction between PA and TXx had the most significant impact on the spatial differentiation of grassland NPP, with an explanatory power of 0.53. This further confirmed the dominant role of PA in driving grassland NPP change in Tajikistan. Moreover, the synergistic effects between different seasonal extreme climate events enhanced the spatial differentiation of grassland NPP. The interactions influencing grassland NPP spatial differentiation varied across different altitudinal bands: in areas below 500 m, grassland NPP changes were driven primarily by the interaction between ELD and TR20, which had an explanatory power of 0.66; in the 500–1000 m band, the interaction between PA and CDD influenced grassland NPP spatial differentiation with an explanatory power of 0.76; in the 1000–2000 m band, the interaction between PA and SDII was the primary driver, with an explanatory power of 0.63; and in the 2000–3000 m and >3000 m bands, the interactions between PA and TNx and between CWD and R20 were the primary factors influencing grassland NPP spatial differentiation, with explanatory powers of 0.51 and 0.69, respectively.

### 3.4. Time-Lag Effect of Extreme Climate Indices on Grassland Vegetation

**Figure 9:**
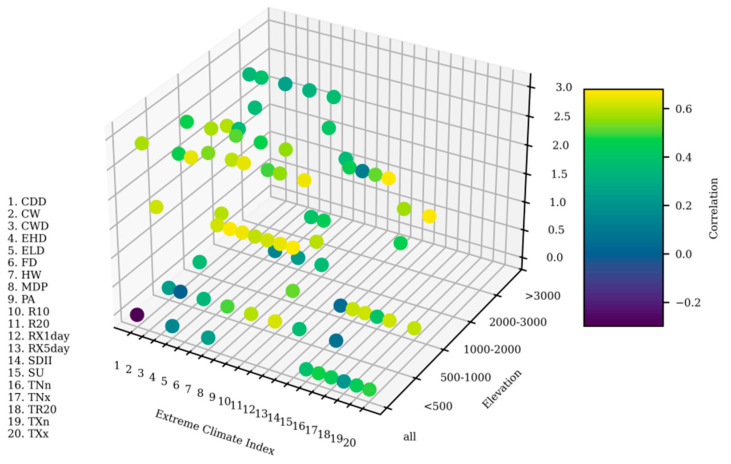
Time-lag between extreme climate indices and grassland vegetation NPP across different altitude gradients.

The time-lag in the effect of seasonal extreme climate events on grassland vegetation revealed significant correlations between extreme climate indices and grassland NPP over a 0–3-month timescale. Different extreme climate indices exhibited varying lag times in their influence on the grassland NPP across Tajikistan (Figure 9). The indices of TXx, TNn, TNx, and TXn showed significant synchronous effects on grassland NPP at the national scale. Conversely, R10, RX1day, RX5day, SDII, CWD, R20, MDP, and PA all exhibited highly significant pronounced two-month time-lags in their effects. CW and FD displayed highly significant three-month time-lags in their effects, while ELD had a significant three-month time-lag in its effect. The lag times of the effects of extreme climate indices on grassland NPP varied across altitudinal bands. For instance, FD showed a significant three-month time-lag in its effect on grassland NPP in the 500–1000 m altitude band. PA and SDII exhibited significant two-month time-lags in their effects on grassland NPP in the 1000–2000 m altitude band, while PA demonstrated a similar two-month time-lag in its effect in the 2000–3000 m altitude band.

## 4. Discussion

### 4.1. Contextualizing Our Findings with Previous Work

Our study revealed that, from 2001 to 2022, the NPP of Tajikistan’s grasslands increased at an average rate of 0.01 gC/m^2^ per year. This is consistent with the findings of Umuhoza et al. [34]. Rising extreme temperatures are likely the primary driver of this growth, and they have also accelerated glacier melting and snowmelt and increased precipitation. Together, these factors have significantly enhanced grassland productivity. Additionally, our study highlights significant variation in the NPP of Tajikistan’s grasslands across different elevation bands, which aligns with the conclusions of Bi et al. [35]. These differences are likely influenced by variations in ecological adaptability, vegetation composition, and soil characteristics. In regions below 500 m, grassland NPP was relatively low and showed a declining trend. This decline can be attributed to frequent human activity, high temperatures, and limited precipitation, all of which significantly reduce soil moisture. Furthermore, the increase in evaporation driven by global warming has exacerbated this phenomenon, leading to grassland degradation and reduced productivity in these low-altitude areas. In contrast, grassland NPP was relatively high in mid-latitude regions (500–3000 m), and it exhibited an increasing trend overall. This can be attributed to the dominance of mountains and plateaus in these areas, which support diverse grassland vegetation. Favorable climatic conditions and relatively abundant precipitation further provide an optimal environment for grassland growth and productivity [36]. However, in high-altitude regions (>3000 m), grassland vegetation exhibited a declining trend, similar to that observed in low-altitude areas (<500 m). This decline was likely due to thin, nutrient-poor soils as well as extreme climatic conditions that severely constrain vegetation growth such as low temperatures and intense ultraviolet radiation. Additionally, soil freeze–thaw cycles and limited water availability can further exacerbate a decline in productivity. In our study on the spatiotemporal variation in grassland NPP across Tajikistan and its driving factors, our findings emphasize the differing impacts of climate change and human activity across varying elevation bands. These results provide a scientific basis for the conservation and sustainable management of grassland ecosystems.

Seasonal extreme climate events, which are characterized by their abrupt onset, significant destructiveness, and unpredictability, profoundly impact the carbon cycle of regional terrestrial ecosystems. These events disrupt vegetation growth cycles, influence productivity and yield, and pose substantial challenges to ecosystem stability and functionality [37,38,39,40]. Our study found that extreme temperatures in Tajikistan’s grasslands have exhibited an upward trend since the beginning of the 21st century, while extreme precipitation has decreased slightly (Figure 5). These findings align with the conclusions of Feng et al. [41], further indicating that Tajikistan’s climate has become drier due partly to reduced precipitation since the early 21st century. Our study found that the coefficients of variation for CW, FD, and ELD were relatively high in low-altitude regions. This may be attributed to climate change intensifying seasonal temperature fluctuations, particularly in winter, which leads to greater interannual variability in the frequency and intensity of FD and ELD occurrences. Additionally, agricultural development and land use changes in low-altitude areas may alter local climate patterns. The high variability in CDD in the mid-altitude regions (1000–3000 m) of Tajikistan is likely due to the region’s complex topography, characterized by mountains and plateaus, which creates localized climatic conditions that amplify fluctuations in dry periods. Additionally, human-initiated activities, such as overgrazing, further contribute to the increased variability of CDD. Together, these factors lead to significant fluctuations in consecutive [42]. Our study also explored the spatiotemporal variation of seasonal extreme climate events in Tajikistan and its driving factors. Therefore, we can provide a scientific basis for understanding the dynamics of seasonal extreme climate events.

### 4.2. Driving Factors of Seasonal Extreme Climate Events on Grassland Vegetation in Tajikistan

Studies have shown that seasonal extreme climate events significantly impact vegetation growth, potentially promoting it or inhibiting it. Arid regions where extreme precipitation directly affects soil water availability are particularly vulnerable [43]. In this study, we found that the response of Tajikistan’s grassland NPP to extreme precipitation was significantly stronger than its response to extreme temperature indices. Specifically, PA was identified as the primary factor influencing NPP variation in Tajikistan’s grasslands (Figure 7). This finding is consistent with research by Yao et al. [44] and Yu et al. [45], which further underscores the crucial role of extreme precipitation, especially for continuous concentrated precipitation in driving vegetation productivity in Tajikistan and the broader arid regions of Central Asia. The response of grassland NPP to extreme precipitation varied across different altitude bands. In regions above 3000 m, CWD emerged as the primary factor driving changes in grassland NPP. This phenomenon can be attributed to elevation-dependent wetting enhancement (EDWE), where the impact of extreme precipitation is altitude-dependent [46]. Hu et al. [47] previously emphasized that EDWE is primarily reflected in extreme precipitation indices such as R10 and CWD, while other indices do not exhibit this altitude dependence. The variation observed here was likely due to the complex precipitation mechanisms in Tajikistan and its surrounding regions. We also found that annual precipitation was the main factor driving NPP variation in the grasslands of Tajikistan at elevations below 3000 m. This may be due to the relatively higher temperatures and lower precipitation in lower-altitude areas, where excessive rainfall can reduce soil organic matter and disrupt grassland growth. Precipitation directly affects the availability of soil moisture, and thus changes in precipitation lead to significant variation in water resource availability. Fittingly, the influence of annual precipitation on grassland growth was more pronounced in mid-elevation areas. This finding aligns with the conclusions drawn in the studies by Guo et al. [48] and Ma et al. [49].

Vegetation growth is a continuous and dynamic process constrained by certain factors. Interactions between two factors significantly influenced the spatial heterogeneity of grassland NPP in Tajikistan (Figure 8). Notably, the interaction between the extreme precipitation index PA and the extreme temperature index TXx had a pronounced impact, which indicates that the primary drivers of Tajikistan’s grassland NPP are the combined effects of extreme precipitation and temperature events. This water–heat synergy was dominant in influencing grassland growth in the study area. In low-altitude regions, the primary interaction was between ELD and TR20. This difference can be attributed to relatively higher temperatures in these areas, with ELD likely affecting physiological processes such as growth cycles, germination, and flowering. Simultaneously, TR20 events may lead to water accumulation, which can result in soil erosion and root hypoxia. Therefore, the combined impact of these factors significantly influences grassland growth in low-altitude areas [50,51].

### 4.3. Time-Lag Effects of Seasonal Extreme Climate Events on Grassland Growth

Both extreme temperatures and precipitation influenced grassland growth in the study area, though with distinct regional variations. Grassland NPP exhibited a clear, time-lagged response to these seasonal extreme climate events. This study demonstrates that Tajikistan’s grassland NPP responds to seasonal extreme climate events at a lag time ranging from zero to three months, with the primary responses occurring in the same month or within two months. Among the 11 extreme temperature indices analyzed, 8 indices showed a 0-month lag in their effect on grassland NPP growth. In one example, an increase in temperature accelerated the onset of the growing season, with its effects becoming evident within the same month. In summary, temperature changes typically have an immediate and direct impact on grassland growth. Consistent with the findings of Sumner et al. [52], we also observed that the effects of the CW, ELD, and FD indices on grassland growth had a 3-month lag time. This may be attributed to extreme cold events that contributed to a reduction in soil temperature, potentially freezing the soil, and thereby suppressing root growth and water absorption. This phenomenon directly affects the vegetative growth cycle. Among the nine extreme precipitation indices, eight (all except for CDD) showed a 2-month lag in their effect on grassland growth. This may be because grasslands in arid and semiarid regions face significant water stress. After experiencing drought or periods of water scarcity, extreme precipitation may promote grassland recovery and growth. However, this recovery requires time, and the effects of precipitation on grassland growth are not immediate, especially because it takes time for water to infiltrate the soil and be absorbed by the vegetation [53,54].

Across Tajikistan, the effect of PA on grassland NPP had a significant lag, with the longest lag time extending up to 3 months. This can be attributed to the typical shallow-rooted grassland vegetation, with its growth primarily dependent on surface soil moisture. FD exhibited an effect on grassland NPP after a 3-month lag in regions below 500 m and between 500–1000 m, with the effect being particularly pronounced in the 500–1000 m range. This was likely due to frost reducing soil temperatures, suppressing root activity, and slowing water absorption [55]. These impacts require extended periods to dissipate, so they influence grassland growth gradually. In the 1000–3000 m altitude range, PA showed a significant effect on grassland NPP after a 2-month lag. Extreme precipitation events directly affect soil moisture, a crucial factor for grassland growth. However, the ecosystem’s response time is prolonged due to the relatively slow rate at which grasslands absorb water, especially in medium-to-high-altitude regions. Our study emphasizes the time-lag of the effects of seasonal extreme climate events on the NPP of grassland vegetation in Tajikistan. These findings highlight the importance of formulating more comprehensive management strategies that consider both the immediate and the delayed ecosystem responses to seasonal extreme climate conditions across varying altitudes.

### 4.4. Uncertainties

Our analysis of climate lag effects on Tajikistan’s grassland NPP acknowledges certain limitations in model parameterization, though our sensitivity analyses provide essential context (Figure S1). While the maximum light energy utilization (ε) parameters derived from established literature may introduce regional uncertainties in absolute NPP estimates [56,57], our comprehensive sensitivity testing (ε = 0.8–1.1) demonstrates that the identified lag-time patterns remain remarkably stable despite potential parameter variations. Furthermore, NPP magnitude exhibits only moderate sensitivity (0–10%) to these variations (Figure S2). However, our current climate-centric framework does not account for anthropogenic influences, such as grazing pressure, which may interact with climatic responses. These findings suggest that while precise NPP quantification requires careful parameter calibration, the underlying temporal response patterns remain robust. This provides a solid foundation for climate impact assessments, which future studies can further refine by incorporating human-driven factors and optimizing region-specific parameters.

## 5. Conclusions

We analyzed the lag effects of seasonal extreme climate events on grasslands at different altitude gradients in Tajikistan between 2001 and 2022. The main findings of this study are as follows:

1. Over the past 22 years, the grassland NPP in Tajikistan has shown a slightly increasing trend [0.01 gC/(m^2^·a)]. Most extreme temperature indices have shown a growing trend, with TXn, TNn, TR20, and HW all showing significant upward trends (*p* < 0.01). Most extreme precipitation indices have shown a decreasing trend (except for CDD), with CDD and CWD exhibiting significant trends;
2. In low-altitude regions, the interaction between extreme drought and high-temperature events has a significant impact on grassland productivity. In contrast, in mid-to high-altitude areas, extreme temperature events, particularly low-temperature events, can inhibit grassland photosynthesis and growth cycles even though precipitation is relatively abundant;
3. In low-altitude areas, the lag time of the effect of seasonal extreme climate events on grassland NPP ranges from 2 to 3 months, while in mid-altitude areas, the lag time is primarily 2 months. Additionally, we found that the lag time of the effect of FD on grassland NPP was 3 months in areas at altitudes of <500 m, 500–1000 m, and 2000–3000 m. Similarly, the lag time of the effect of PA on grassland NPP was a significant 2 months (*p* < 0.05) in the 1000–3000 m elevation bands.

## Figures and Tables

**Table 1:** Description of precipitation and temperature indices.

Index Types	Descriptive Name	Indices	Definitions
Extreme temperature indices	Frost days	FD	Daily minimum temperature < 0 °C per month
	Summer days	SU	Daily maximum temperature > 25 °C per month
	Min Tmin	TNn	The minimum daily minimum temperature per month
	Min Tmax	TXn	The minimum daily maximum temperature per month
	Max Tmin	TNx	The maximum daily minimum temperature per month
	Max Tmax	TXx	The maximum daily maximum temperature per month
	Hot nights	TR20	Daily maximum temperature > 25 °C per month
	Heatwaves	HW	Daily maximum temperature > 35 °Cper month
	Cold waves	CW	Daily minimum temperature < −10 °C per month
	Extreme hot days	EHD	Daily maximum temperature > 40 °C per month
	Extreme cold days	ELD	Daily minimum temperature < −20 °C per month
Extreme precipitation indices	Maximum one day precipitation	RX1day	Maximum daily precipitation per month
	Maximum five-day precipitation	RX5day	Maximum 5-day consecutive precipitation
	Precipitation anomaly	PA	Daily precipitation-annual average precipitation
	Max precipitation	MDP	Maximum daily precipitation
	Heavy precipitation	R10	Daily precipitation > 10 mm per month
	Very heavy precipitation	R20	Daily precipitation > 20 mm per month
	Consecutive dry	CDD	Daily precipitation < 1 mm per month
	Consecutive wet	CWD	Daily precipitation ≥ 1 mm per month
	Simple daily intensity index	SDII	Monthly total precipitation/precipitation days

**Table 2:** Interaction relationships.

Interaction	Description
Weaken and nonlinear	q(x_1_ ∩ x_2_) < min(q(x_1_), q(x_2_))
Weaken and univariate	min(q(x_1_), q(x_2_)) < q(x_1_∩x_2_) < max(q(x_1_), q(x_2_))
Enhance and bivariate	q(x_1_ ∩ x_2_) > max(q(x_1_), q(x_2_))
Independent	q(x_1_ ∩ x_2_) = max(q(x_1_), q(x_2_))

## Data Availability

Data are contained within the article and Supplementary Materials.

## Supplementary Materials

The following supporting information can be downloaded at https://www.mdpi.com/article/10.3390/plants14081266/s1. Figure S1. Lag effects of grassland NPP under extreme climate index scenarios. Figure S2. Systematic sensitivity analysis of the ε parameter.
