# Supplementary material for: Time-Lag of Seasonal Effects of Extreme Climate Events on Grassland Productivity Across an Altitudinal Gradient in Tajikistan
Source: Plants (Basel). 2025 Apr 21;14(8):1266. doi: 10.3390/plants14081266 (PMC12030477; doi:10.3390/plants14081266)
Supplement: Supplementary file 1 [file plants-14-01266-s001.zip › plants-3555308-supplementary.pdf]

plants

MDPI

Article

Time-Lag of Seasonal Effects of Extreme Climate Events on Grassland Productivity Across an Altitudinal Gradient in Tajikistan

Yixin Geng [1,2,3], Hikmat Hisoriev [4], Guangyu Wang [1,2,3], Xuexi Ma [1,2], Lianlian Fan [1,2], Okhonniyozov Mekhrovar [1,2], Madaminov Abdullo [4], Jiangyue Li [1,2,*] and Yaoming Li [1,2,3,*]

1 Key Laboratory of Ecological Safety and Sustainable Development in Arid Lands, Xinjiang Institute of Ecology and Geography, Chinese Academy of Sciences, Urumqi 830011, China; gengyixin23@mails.ucas.ac.cn (Y.G.); wangguangyu19@mails.ucas.ac.cn (G.W.); maxx@ms.xjb.ac.cn (X.M.); flianlian@ms.xjb.ac.cn (L.F.); okhonniyozov.mekhrovar@gmail.com (O.M.)
2 Research Center for Ecology and Environment of Central Asia, Chinese Academy of Sciences, Urumqi 830011, China
3 University of Chinese Academy of Sciences, Beijing 100049, China
4 Institute of Botany, Plant Physiology and Genetics of Tajikistan Academy of Sciences, Dushanbe 734002, Tajikistan; hhikmat@mail.ru (H.H.); asrorijon@mail.ru (M.A.)
* Correspondence: lijiangyue@ms.xjb.ac.cn (J.L.); lym@ms.xjb.ac.cn (Y.L.)

Sensitivity analysis

To assess the robustness of grassland NPP's lagged response to climate change, this study systematically conducted sensitivity analyses under seven scenarios, including extreme climate index variations (±10%, ±30%, ±50%) and an unchanged baseline scenario. As shown in Figure S1, grassland NPP exhibited remarkable stability in lag time across a wide range of climate index variations (±10% to ±50%), demonstrating consistent patterns despite varying climatic perturbations.

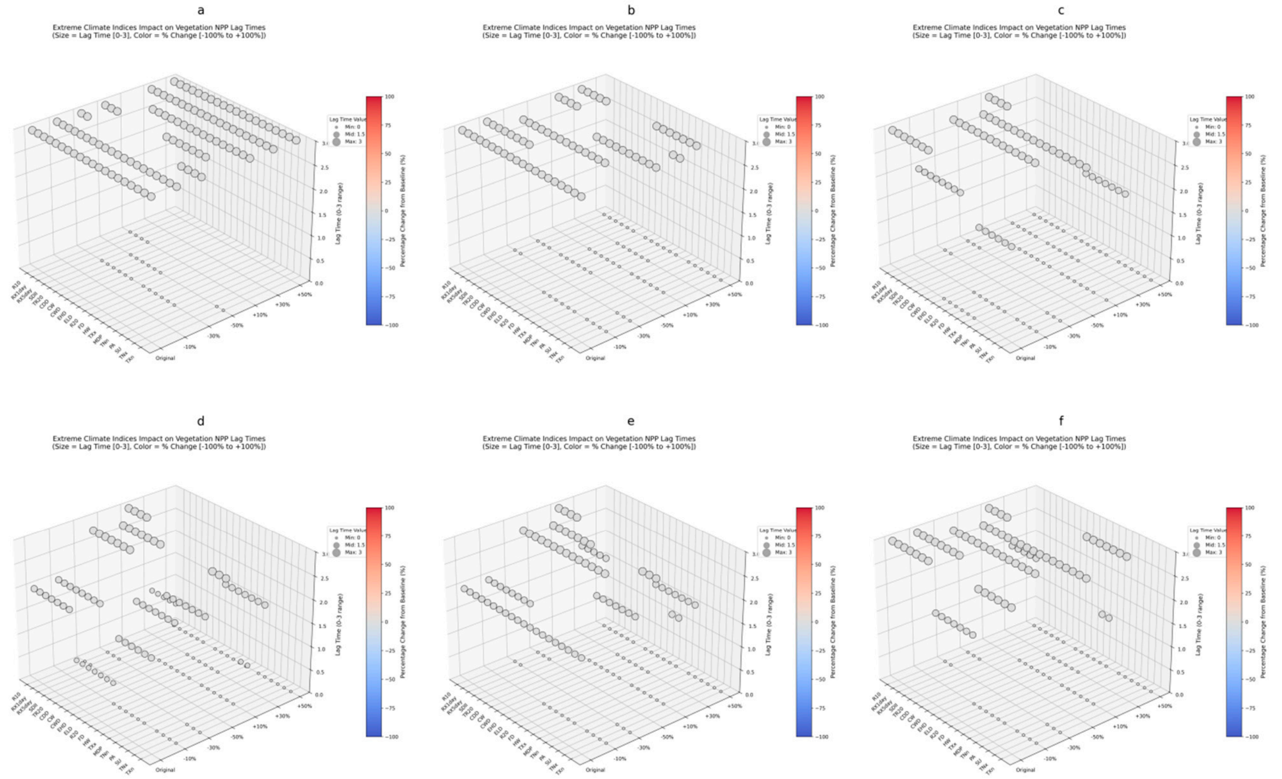

Figure S1 Lag effects of grassland NPP under extreme climate index scenarios. a, entire Tajikistan; b, elevation <500 m; c, elevation 500-1000 m; d, elevation 1000-2000 m; e, elevation 2000-3000 m; f, elevation >3000 m.

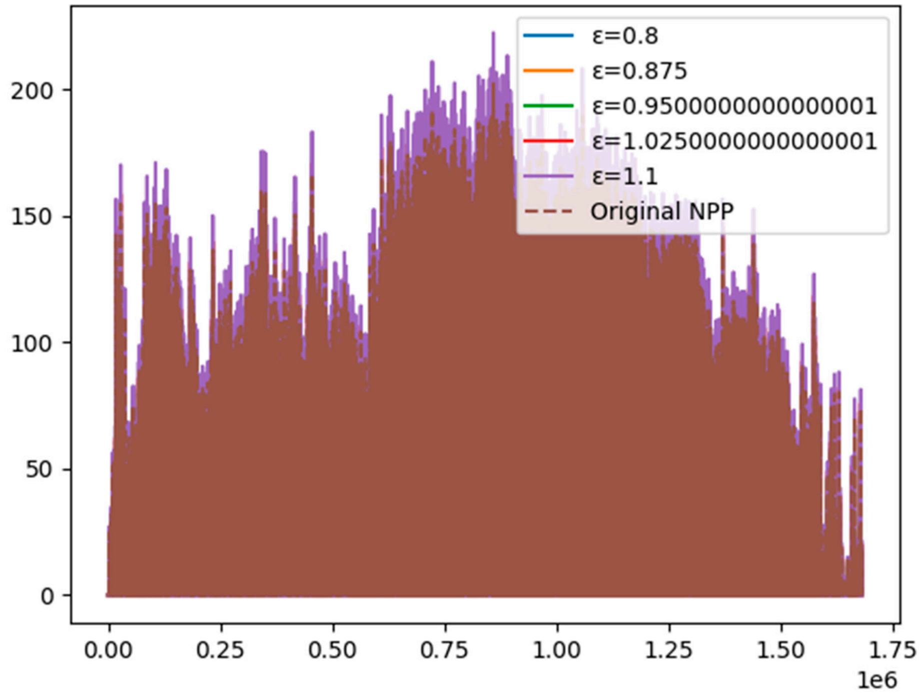

Figure S2 Systematic sensitivity analysis of the ε parameter.
